# A previously uncharacterized gene *stm0551* plays a repressive role in the regulation of type 1 fimbriae in *Salmonella enterica* serotype Typhimurium

**DOI:** 10.1186/1471-2180-12-111

**Published:** 2012-06-20

**Authors:** Ke-Chuan Wang, Yuan-Hsun Hsu, Yi-Ning Huang, Kuang-Sheng Yeh

**Affiliations:** 1 Graduate Institute of Medical Sciences, College of Medicine, Taipei Medical University, Taipei, Taiwan; 2 Department of Microbiology and Immunology, School of Medicine, College of Medicine, Taipei Medical University, Taipei, Taiwan; 3 Department of Veterinary Medicine, School of Veterinary Medicine, College of Bioresources and Agriculture, National Taiwan University, Taipei, Taiwan

**Keywords:** *Salmonella enterica* serotype Typhimurium, Type 1 fimbriae, c-di-GMP, Phosphodiesterase

## Abstract

**Background:**

*Salmonella enterica* serotype Typhimurium produces surface-associated fimbriae that facilitate adherence of the bacteria to a variety of cells and tissues. Type 1 fimbriae with binding specificity to mannose residues are the most commonly found fimbrial type. *In vitro*, static-broth culture favors the growth of *S.* Typhimurium with type 1 fimbriae, whereas non-type 1 fimbriate bacteria are obtained by culture on solid-agar media. Previous studies demonstrated that the phenotypic expression of type 1 fimbriae is the result of the interaction and cooperation of the regulatory genes *fimZ*, *fimY*, *fimW,* and *fimU* within the *fim* gene cluster. Genome sequencing revealed a novel gene, *stm0551*, located between *fimY* and *fimW* that encodes an 11.4-kDa putative phosphodiesterase specific for the bacterial second messenger cyclic-diguanylate monophosphate (c-di-GMP). The role of *stm0551* in the regulation of type 1 fimbriae in *S*. Typhimurium remains unclear.

**Results:**

A *stm0551*-deleted stain constructed by allelic exchange constitutively produced type 1 fimbriae in both static-broth and solid-agar medium conditions. Quantative RT-PCR revealed that expression of the fimbrial major subunit gene, *fimA,* and one of the regulatory genes, *fimZ*, were comparably increased in the *stm0551*-deleted strain compared with those of the parental strain when grown on the solid-agar medium, a condition that normally inhibits expression of type 1 fimbriae. Following transformation with a plasmid possessing the coding sequence of *stm0551*, expression of *fimA* and *fimZ* decreased in the *stm0551* mutant strain in both culture conditions, whereas transformation with the control vector pACYC184 relieved this repression. A purified STM0551 protein exhibited a phosphodiesterase activity *in vitro* while a point mutation in the putative EAL domain, substituting glutamic acid (E) with alanine (A), of STM0551 or a FimY protein abolished this activity.

**Conclusions:**

The finding that the *stm0551* gene plays a negative regulatory role in the regulation of type 1 fimbriae in *S.* Typhimurium has not been reported previously. The possibility that degradation of c-di-GMP is a key step in the regulation of type 1 fimbriae warrants further investigation.

## Background

*Salmonella* species are some of the most important food-borne pathogens in the world. Members of the genus *Salmonella* are gram-negative, facultative anaerobic rods which are composed of more than 2500 serotypes [1]. *Salmonella enterica* serotype Typhimurium (*S*. Typhimurium) is an important causative agent for gastroenteritis. For most bacteria, adhesion to host epithelial cells is the first step in establishing an infection. Adhesion proteins or hair-like appendages called fimbriae on the outer membranes of bacteria have been implicated in adherence [2]. Whole-genome sequencing identified 13 separate fimbrial gene clusters that may have the potential to encode fimbria-associated proteins in *S*. Typhimurium [3]. Among these, type-1 fimbriae are the most commonly found type in *S*. Typhimurium, as in other members of the family *Enterobacteriaceae*[4]. In addition to adherence, type 1 fimbriae also contribute to virulence and biofilm formation [5-7].

Phenotypic expression of type 1 fimbriae in *S*. Typhimurium involves the interaction and cooperation of genes in the *fim* gene cluster. Briefly, FimA, FimI, FimF, and FimH are structural proteins that are incorporated to assemble a fimbrial shaft structure, while FimC and FimD proteins located in the periplasmic space and on the outer membrane respectively, function to transport and anchor the fimbrial proteins. FimZ, FimY, FimW, and an arginine transfer RNA *fimU*, regulate fimbrial production by a complicated network [8-12]. Studies also demonstrated that a global regulator, leucine-responsive regulatory protein (Lrp), and other genes outside the *fim* gene cluster also take part in the regulatory expression of type-1 fimbriae [13,14].

**Figure 1:**
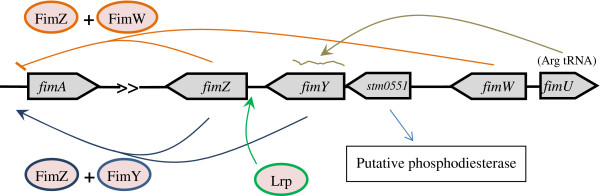
**The genetic organization of the *S* . Typhimurium *fim* gene cluster and a possible regulatory network.** The predicted sizes of the Fim polypeptides are given in kilodaltons (kDa) with Arabic numbers. The arrows indicate the direction of transcription. The signal peptide region of each gene product is shown as a small filled box. The established or postulated functions of the genes are indicated as follows: *fimA*, major fimbrial subunit; *fimI*, minor fimbrial subunit; *fimC*, chaperone protein; *fimD*, molecular usher; *fimH*, adhesion protein; *fimF*, minor fimbrial subunit; *fimZ*, regulatory protein; *fimY*, regulatory protein; *stm0551*, phosphodiesterase; *fimW*, regulatory protein; *fimU*, arginine tRNA. FimZ and FimY are both required to activate *fimA*. FimW represses *fimA* expression and FimW interacts with FimZ physically to consume FimZ, diminishing available FimZ to activate *fimA*. Leucine-responsive regulatory protein (Lrp) activates *fimZ*. *fimU* activate FimY translation. The function of *stm0551* within the *fim* regulatory circuit needs further characterization.

**Figure 2:**
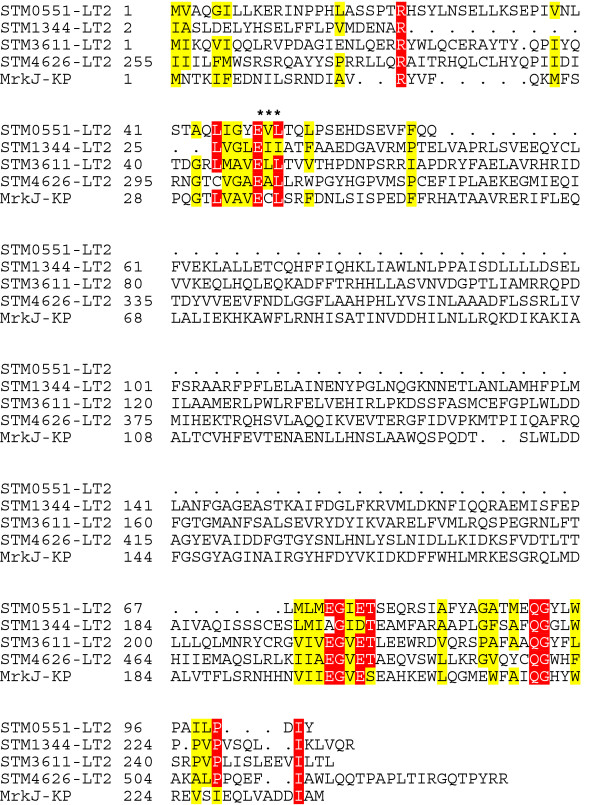
**Multiple sequence alignment of the EAL domain of STM0551 and other experimentally studied proteins.** Residues showing strict identity are written in white characters and highlighted in red. Similarity across groups is indicated with black characters and highlighted in yellow. Putative catalytic active site residues within the EAL domain are marked with an asterisk. Protein names and microorganisms are as follows: STM0551, STM1344, STM3611, STM4264: *S*. Typhimurium LT2; MrkJ: *K*. *pneumoniae.*

Bis-(3′–5′)-cyclic dimeric GMP (c-di-GMP) is a universal second messenger that controls cell surface-associated characters in bacteria [15]. Recent studies revealed the importance of c-di-GMP in regulating many physiological process such as adhesion, biofilm formation, exopolysaccharide synthesis, virulence, and motility [16,17]. The cellular c-di-GMP concentration is regulated by diguanylate cyclase (DGC) and phosphodiesterase (PDE). DGC catalyzes the formation of c-di-GMP through a linear intermediate, pppGpG, while PDE degrades it into guanosine monophosphate (GMP). The most prominent conserved protein domains in the PDE are EAL and HD-GYP [17]. An open reading frame named *stm0551*, located between *fimY* and *fimW*, has not previously been investigated to determine its involvement in type-1 fimbrial regulation in *S*. Typhimurium (Figure 1). The amino acid sequence of the STM0551 protein could encode a putative PDE. Multiple alignments of the EAL domain of STM0551 with other known PDE enzymes demonstrated the preservation of several regions throughout the domain sequence >(Figure 2). Since STM 3611 influences curli fimbrial expression in *S*. Typhimurium, and MrkJ controls type 3 fimbriae production and biofilm formation in *Klebsiella pneumoniae*[18,19], we decided to investigate whether *stm0551* encodes a functional PDE that plays a role in type 1 fimbrial expression.

In the present study, a *stm0551* mutant was constructed by allelic exchange. Phenotypic and genotypic characteristics of this mutant were analyzed. Purified STM0551 protein was tested for its putative function as a PDE *in vitro*. A possible role of *stm0551* in type 1 fimbrial regulation in *S*. Typhimurium is discussed.

## Results

### Type 1 fimbrial expression by the *S*. Typhimurium *stm0551* mutant strain

**Figure 3:**
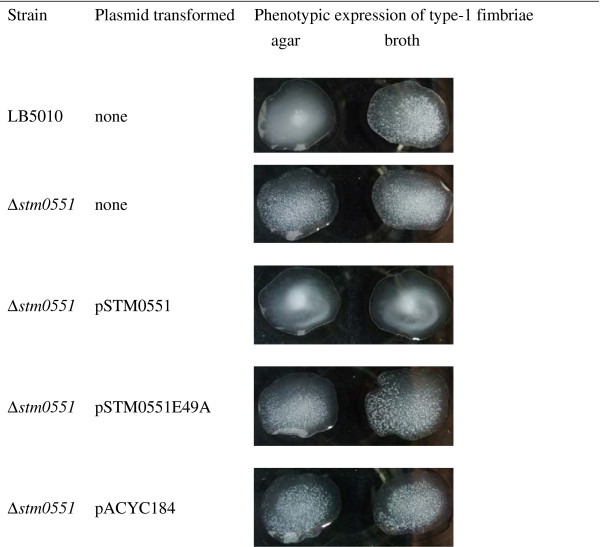
**Phenotypic expression of type 1 fimbriae in *S* . Typhimurium analyzed by yeast agglutination test.***S*. Typhimurium LB5010 prepared from broth medium exhibited positive agglutination phenotype, while those prepared from agar medium showed homogenous appearance on the glass slide. Δ*stm0551* strain, prepared from either agar or broth medium, both demonstrated agglutination. Transforming pSTM0551 into Δ*stm0551* inhibited agglutination. The transformants possessing either pSTM0551E49A or pACYC184 cloning vector exhibited the same agglutination phenotype as Δ*stm0551* strain.

The bacterial strains and plasmids used were described in Table 1, while the primers used was indicated in Table 2. The *S*. Typhimurium *stm0551* knockout mutant strain was constructed by a one-step gene inactivation method [20]. Primers stm0551-F and stm0551-R external to *stm0551* amplified a 0.5-kb DNA fragment from *S*. Typhimurium LB5010 genomic DNA, while the same primer set generated a 1.3-kb DNA fragment from genomic DNA of the *S.* Typhimurium *stm0551* mutant strain, indicating a kanamycin cassette inserted into the *stm0551* gene. This DNA fragment was also sequenced to determine its identity. The confirmed *stm0551* mutant strain was then designated *S*. Typhimurium Δ*stm0551*. *S*. Typhimurium LB5010 mediated yeast agglutination and guinea pig erythrocyte when cultured in static LB broth, whereas agglutination was not detected when cells were collected from LB agar (Table 3). In contrast, the *S*. Typhimurium Δ*stm0551* strain mediated agglutination when grown on LB agar. Nonetheless the degree of agglutination was not as strong as the same strain grown in static LB broth. Transformation of the pSTM0551 plasmid that contains the coding sequence of *stm0551* conferred on *S*. Typhimurium Δ*stm0551* strain the ability to inhibit type 1 fimbrial expression in both culture conditions, while the *S*. Typhimurium Δ*stm0551* strain carrying a plasmid that possessed a *stm0551* coding sequence with the glutamic acid (E) at position 49 replaced with an alanine (A), or a pACYC184 cloning vector exhibited the same phenotype as the *S*. Typhimurium Δ*stm0551* strain. The Figure 3 demonstrated the yeast agglutination tests performed on glass slides.

**Table 1:** Bacterial strains and plasmids used in this study

**Name**	**Description ^a^**	**Reference or source**
*Salmonella enterica* serotype Typhimurium		
LB5010	Wild type *S*. *enterica* serotype Typhimurium LT2 strain, fimbriate with the complete *fim* gene cluster	[21]
Δ*stm0551*	*stm0551* deletion mutant; Kan^r^	Present study
*Escherichia coli* strain		
One Shot® TOP10 chemically competent *E*. *coli*	F^-^*mcr*A Δ(*mrr*-*hsd*RMS-*mcr*BC) Φ80*lacZ*ΔM15 Δ*lac*X74 *rec*A1 *ara*D139Δ(*ara*-*leu*)7697 *gal*U *gal*K *rps*L (Str^R^) *end*A1 *nup*G	Invitrogen
BL21Star™ (DE3) One Shot® chemically competent *E*. *coli*	F^-^*ompT hsdS*_*B*_(r_B_^-^ m_B_^-^) *gal dcm* (DE3)	Invitrogen
Plasmids		
pSTM0551	A 0.5-kb DNA fragment possessing the *stm0551* coding sequence cloned into the pACYC184 vector; Cm^r^	Present study
pSTM0551E49A	A 0.5-kb DNA fragment possessing the *stm0551* coding sequence with glutamic acid (E) at position 49 replaced with an alanine (A) cloned into the pACYC184 vector; Cm^r^	Present study
pACYC184	Tet^r^, Cm^r^, cloning vector; w/p15A *ori*	ATCC, Manassas, VA
pET101/D-TOPO	Amp^r^, 6xHis tag expression vector	Invitrogen
pKD46	Amp^r^, express λ Red recombinase system, temp- sensitive replicon	[22]
pKD13	Plasmid carrying Kan^r^ cassette	[22]

^a^*Amp*^*r*^ ampicillin resistant; *Cm*^*r*^ chloramphenicol resistant; *Kan*^*r*^ kanamycin resistant; *Tet*^*r*^ tetracycline resistant

**Table 2:** Primers used in the present study

**Purpose and name**	**Sequence (5′ to 3′)**	**Description**
Construction of the *stm0551* mutant		
stm0551pKD13-F	GCTCTGATGTTTCAATGCCTTCCATCAGC ATTAACTGATTCCGGGGATCCGTCGACC	Annealing Temp.: 55°C; amplicon length: 1,550 bp
stm0551pKD13-R	GGCACAGGGTATTTTGTTAAAGGAAAGG ATAAATCCCTGTAGGCTGGAGCTGCTTCG	
Cloning		
stm0551-F	GGATCCCATCCTGCTTTTTCCATTGCTCTAATAT	*Bam*HI restriction site (underlined)
stm0551-R	GATATCACTCACTTAACTTTTTACAAGGCTTACG	*Eco*RV restriction site (underlined)
		Annealing Temp.: 55°C; amplicon length: 500 bp
Construction of the fusion protein		
stm0551-TOPO-F	CACCATGGTGGCACAGGGTATTTTGTTAA	Annealing Temp.: 50°C; amplicon length: 316 bp
stm0551-TOPO-R	ATATATATCTGGTAATATGGCTGG	
fimY-TOPO-F	CACCATGCGCAGCGTACCACGCAG	Annealing Temp.: 50°C; amplicon length: 727 bp
fimY-TOPO-R	AAAAATGTCGTGGAAAGTAACGT	
E49A-TOPO-F	ATCGGCTATGCGGTCCTGACGCAACTTCCG	Mutation site (underlined)
E49A-TOPO-R	CGGAAGTTGCGTCAGGACCGCATAGCCGAT	Mutation site (underlined)
RT-PCR analysis		
fimA-RT-F	ACTATTGCGAGTCTGATGTTTG	
fimA-RT-R	CGTATTTCATGATAAAGGTGGC	
fimZ-RT-F	ATTCGTGTGATTTGGCGT	
fimZ-RT-R	ACTTATCCTGTTGACCTT	
fimY-RT-F	GAGTTACTGAACCAACAGCT	
fimY-RT-R	GCCGGTAAACTACACGATGA	
fimW-RT-F	AAAGTGAAAGTAAAGCGG	
fimW-RT-R	AAGAGATAGATAATGCCCG	
stm0551-RT-F	GCCATAAATAACCTTGTTCC	
stm0551-RT-R	CATTCATATCTCAACAGCGA	
16 s-F	TTCCTCCAGATCTCTACGCA	
16 s-R	GTGGCTAATACCGCATAACG	

**Table 3:** **Phenotypic expression of type 1 fimbriae in *S* . Typhimurium**

**Strain**	**Plasmid transformed**	**Phenotypic expression of type-1 fimbriae ^a^**	
		agar	broth
LB5010	none	-	++
Δ*stm0551*	none	+	++
Δ*stm0551*	pSTM0551	-	-
Δ*stm0551*	pSTM0551E49A	+	++
Δ*stm0551*	pACYC184	+	++

^a^ Phenotypic expression of type-1 fimbriae was determined using a mannose-sensitive yeast agglutination test and guinea pig erythrocyte hemagglutination test

### Electron microscopy

**Figure 4:**
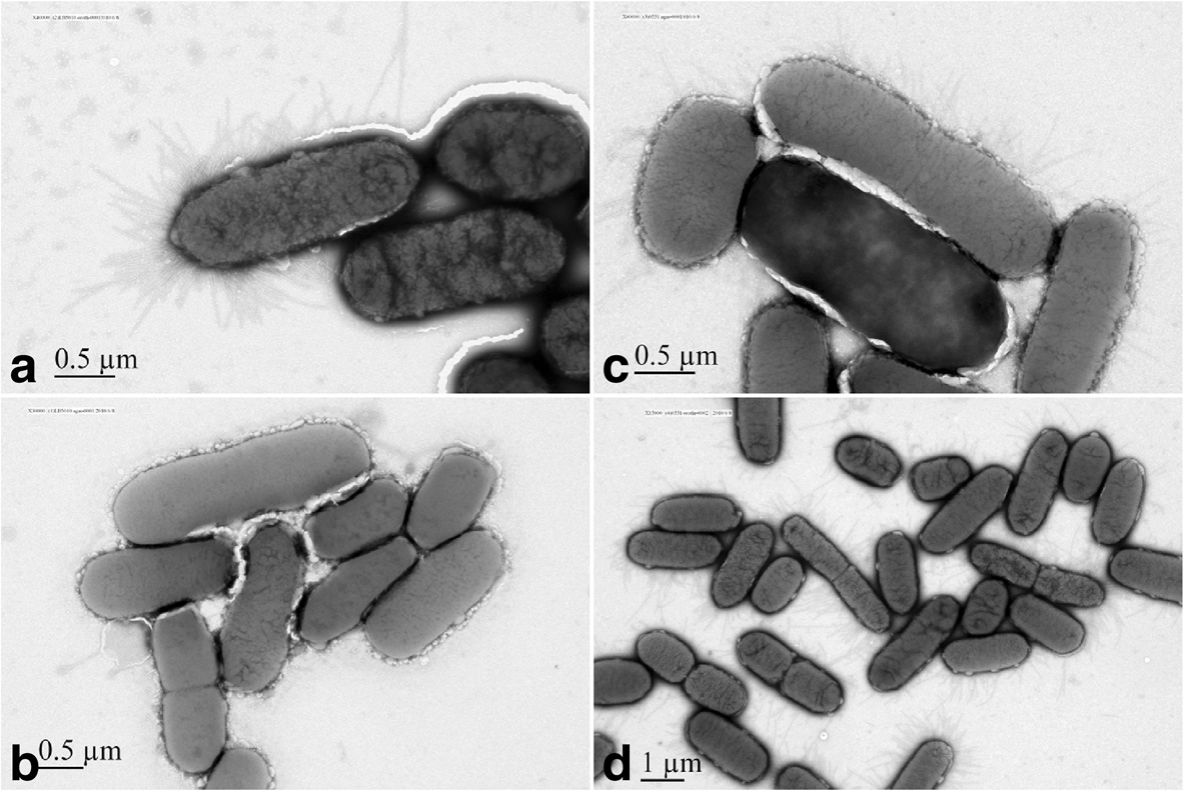
**Observation of *S* . Typhimurium LB5010 and the *S* . Typhimurium Δ *stm0551* strain by electron microscopy.** Panel A: *S*. Typhimurium LB5010 obtained following growth under static LB broth conditions at 37°C for 48 h produced type 1 fimbrial appendages (40,000 ×). Panel B: No fimbrial structures were observed on the *S*. Typhimurium LB5010 grown on LB agar at 37°C for 18 hr (30,000 ×). Panel C: *S*. Typhimurium Δ*stm0551* cells obtained from static LB broth condition at 37°C for 48 h produced type 1 fimbrial appendages (15,000 ×) whereas the fimbrial appendages were also observed when these cells were cultured on LB solid agar (panel D) (40,000 ×). Bacterial cells were negatively stained with 2% phosphotungstic acid.

*S*. Typhimurium LB5010 prepared in static LB broth culture demonstrated fimbrial appendages on the outermembrane of the cell (Figure 4, panel A). On the contrary, *S*. Typhimurium LB5010 grown on agar medium did not produce type1 fimbriae (Figure 4, panel B). The *S*. Typhimurium Δ*stm0551* strain prepared from static broth medium (Figure 4, panel C) or agar (Figure 4, panel D) produced fimbrial structures.

### Quantitative RT-PCR analysis

**Figure 5:**
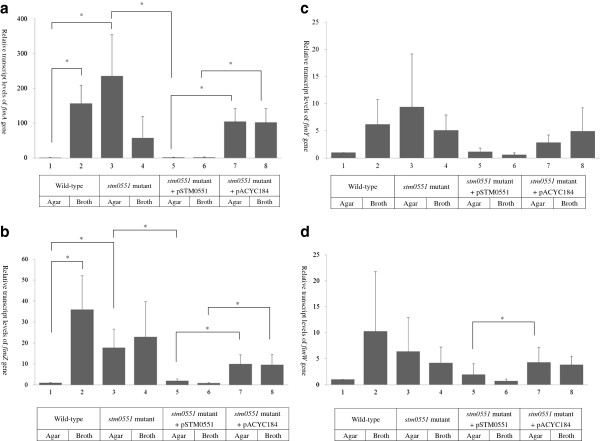
**Detection of the relative transcript levels of *fimA* , *fimZ* , *fimY* , and *fimW* genes using quantitative RT-PCR.** The mRNA transcript levels of the major fimbrial subunit gene *fimA* (panel A), *fimZ* (panel B), *fimY* (panel C), and *fimW* (panel D) in the parental LB5010, Δ*stm0551*, Δs*tm0551* (pSTM0551), and Δ*stm0551* (pACYC184) strains were detected by quantitative RT-PCR. The mRNA transcript levels were obtained by delta-delta Ct (ΔΔCt) method, and the expression levels (2^-ΔΔCt^) of the parental LB5010 strain cultured on LB agar were set to 1 fold for each gene tested. The asterisk indicated that the differences in transcript levels were statistically significantly (*p*<0.05).

Total RNA from LB5010, Δ*stm0551*, Δ*stm0551* (pSTM0551), and Δ*stm0551* (pACYC184) strains were prepared and analyzed for the fimbrial subunit gene, *fimA*, and the regulatory genes, *fimZ*, *fimY*, and *fimW,* by quantitative RT-PCR. 16 S ribosomal (r) RNA expression was used as a control. Individual gene expression profiles were first normalized against the 16 S rRNA gene and then compared to the expression level of *fimA*, *fimZ*, *fimY*, and *fimW* obtained from agar. As for the parental LB5010 strain, *fimA* expression obtained from static LB broth was about 150-fold higher than the value obtained from LB agar. The *fimA* expression of the Δ*stm0551* strain grown on agar was significantly higher than that of LB5010 grown on agar. Transformation of Δ*stm0551* with a plasmid possessing the *stm0551* coding sequence repressed *fimA* expression whether this strain was cultured on agar or in static broth, whereas transformation of the same bacterial strain with the plasmid cloning vector pACYC184 de-repressed *fimA* expression in both culture conditions (Figure 5, panel A). The *fimZ* expression levels from different strains demonstrated a similar profile to that observed for *fimA*. The parental LB5010 strain exhibited significant elevated level of *fimZ* when grown in broth than on agar. The *fimZ* expression of Δ*stm0551* was higher than that of the parental strain grown on agar. Transforming Δ*stm0551* with pSTM0551 repressed *fimZ* expression on both culture conditions, while transforming Δ*stm0551* with pACYC184 cloning vector de-repressed *fimZ* expression, leading to comparable level of expression as seen in the Δ*stm0551* strain (Figure 5, panel B). However, the expression levels of *fimY* were not significantly different between strains under both growth conditions (Figure 5, panel C). Δ*stm0551*(pACYC184) had higher *fimW* expression than Δ*stm0551*(pSTM0551) did when both strains were culture on agar medium (Figure 5, panel D).

### PDE activity

**Figure 6:**
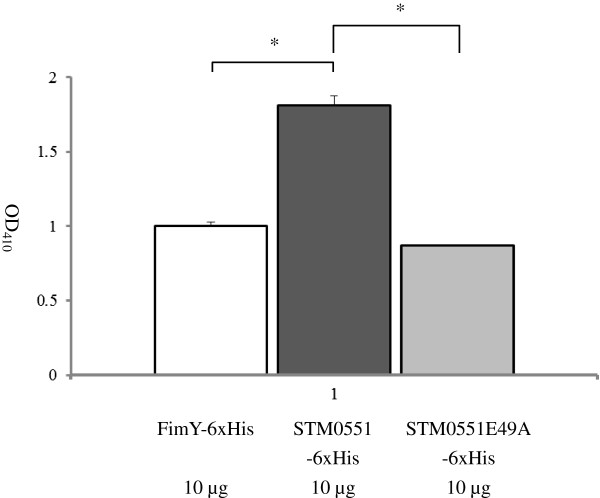
**Phosphodiesterase activity.***In vitro* phosphodiesterase activity assays compared the abilities of the purified STM0551-6xHis, FimY-6xHis, and STM0551E49A-6xHis proteins to cleave the specific substrate, bis (*p*NPP). Release of *p*-nitrophenol was determined at 410 nm. * *p*<0.05.

Since the predicted amino acid sequence of STM0551 suggested that it was related to a family of proteins that exhibit PDE activity, we determined whether STM0551 possessed PDE activity. The *in vitro* PDE activity of the purified STM0551-His fusion protein was determined using the specific substrate, bis (*p*NPP). The purified FimY-His fusion protein was used as a control since FimY amino acids exhibited no domain related to PDE activity. STM0551 possesses “EVL” conserved residues that may form the putative active site that varies from the consensus “EAL” sequence. We constructed a substitution mutation in which the glutamic acid (E) at position 49 was replaced by alanine (A) in the *stm0551* allele. A fusion protein of this construct was prepared with the same procedure described for STM0551 and FimY and was designed as STM0551E49A-His. The reactions that contained STM0551 exhibited a statistically significant 1.75-fold increase in the release of *p*-nitrophenol compared to that containing FimY and STM0551E49A (both reaction mixtures contained the same amount of protein [10 μg]) (Figure 6). This result suggests that STM0551 could function as a PDE.

## Discussion

The regulatory pathway of type 1 fimbriae in *S*. Typhimurium involves several genes including the *fim* gene cluster and other genes such as *lrp*[8-14]. The *Salmonella* pathogenicity island 1 (SPI1) and flagellar systems also crosstalk with type 1 fimbriae [23]. Several studies have indicated that the mechanism controlling the intracellular c-di-GMP concentration plays a critical role in regulating fimbrial production. For example, MrkJ, a PDE, regulates type-3 fimbrial production in *Klebsiella pneumoniae*[19]. Deletion of *mrkJ* resulted in an increase in type-3 fimbrial production [19]. In *Escherichia coli* S fimbriae are regulated by a PDE, SfaY [24]. Production of CupA fimbriae of *Pseudomonas aeruginosa* is controlled by both the GGDEF domain in protein, PA1120, and PvrR that contains an EAL domain [25]. The FimK of *Klebsiella pneumoniae* contains the EAL domain and deletion of *fimK* conferred hyperpiliation of type 1 fimbriae in this bacterium [26]. Our present finding may add one more example to this fimbrial regulation/c-di-GMP concentration circuit.

The *stm0551* gene of *S*. Typhimurium is located within the *fim* gene cluster but has not previously been investigated. The predicted amino acids of STM0551 showed similarity to those of proteins with PDE activity, so it was interesting to further dissect the function of *stm0551* in terms of type 1 fimbrial regulation. The parental strain *S*. Typhimurium LB5010, is an LT2 derivative and displays a variable fimbrial phase [21]. A static broth culture favors *S*. Typhimurium to produce type 1 fimbriae, while non-fimbriae phase bacteria were obtained by growth on solid agar medium [27]. A *stm0551* knockout mutant strain constructed in the present study enabled it to produce type 1 fimbriae on the solid LB agar medium. This phenotype was correlated with the RT-PCR result that the mRNA expression of the major fimbrial subunit, *fimA,* was enhanced on solid-agar culture medium. These suggested that *stm0551* plays a repressive role in type 1 fimbrial regulation perhaps in a similar manner to the role played by FimW in the *fim* regulatory circuit [9]. The expression of *fimA* of the transformant Δ*stm0551* (pSTM0551) grown on agar decreased to the same level as that of the parental LB5010 strain grown in the same conditions. However, this transformant did not exhibit visible yeast agglutination and guinea pig erythrocyte hemagglutination when grown in static broth, nor did this strain exhibit *fimA* expression, which was unexpected. One of the reasons could have been the relatively high level of STM0551 production due to presence of the multiple copies of the pSTM0551 recombinant plasmid in these cells. An excessive STM0551 level in *S*. Typhimurium could presumably cause a dramatically decreased concentration of c-di-GMP locally, and subsequently interfere with *fimA* expression. However, the mechanism by which STM0551 interacts with *fimA* gene expression remains unclear. One possibility is that the *stm0551* product maintained the local concentration of c-di-GMP at a level such that only a certain amount of c-di-GMP was bound by a hypothetical PilZ domain containing protein. This low concentration of c-di-GMP-bound, PilZ domain-containing protein was not able to activate *fimA* gene expression. Disruption of *stm0551* increased the local c-di-GMP concentration and consequently also increased the “functional” PilZ domain-containing protein to enhance *fimA* expression. The FimY protein of *S*. Typhimurium could possibly function as such a PilZ domain-containing protein since recently we found that the amino acid sequence of FimY demonstrated relatedness to those of MrkH of *K*. *pneumoniae* and YcgR of the *E*. *coli* K-12 strain (data not shown). Both MrkH and YcgR were shown to be transcriptional activators with c-di-GMP-binding PilZ domains [28,29]. Our hypothesis about the role FimY correlates with the finding that STM0551 did not affect *fimY* at the transcriptional level (Figure 5, panel C). More detailed study of FimY is necessary to define its role in a possible c-di-GMP regulatory network. Both FimY and FimZ are required to activate *fimA* expression in *S*. Typhimurium [8]. FimZ is a DNA binding protein that binds the *fimA* promoter and activate its expression [30]. Our qRT-PCR results demonstrated very similar profiles for both *fimA* and *fimZ* expression (Figure 5, panel A and B). According to the results reported by Saini *et al.*, FimY and FimZ independently activate the *fimA* gene expression, in addition, FimY and FimZ also activated each other’s expression [31]. Inactivation of *stm0551* could possibly increase the local concentration of c-di-GMP, which results more c-di-GMP bound FimY (active form) available to activate *fimZ* and *fimA*, and eventually amplifies the *fimA* expression. FimW is a repressor for *fimA* in *S*. Typhimurium. FimW may achieve this repressive role by repressing *fimY* transcription or by protein-protein interaction with FimZ [9,31]. In the present study, little information was obtained regarding how *stm0551* may interact with *fimW*.

The purified STM0551 fusion protein possessed the ability to cleave the PDE-specific substrate, bis (*p*NPP), *in vitro*, thus confirming the putative phosphodiesterase function assigned to it in the current databank. The construct STM0551E49A-His contained a point mutation in which the conserved glutamic acid residue at position 49 within the putative active site was replaced with an alanine residue; the STM0551E49A mutant protein was unable to cleave bis (*p*NPP). In accordance with this result, when the STM0551E49A-containg construct cloned into a pACYC184 vector (pSTM0551E49A) was transformed into Δ*stm0551*, the resulting transformant exhibited the same phenotype as that of Δ*stm0551* or Δ*stm0551* possessing pACYC184 cloning vector (Table 3). This further suggested that the glutamic acid at position 49 of STM0551 did play a critical role for phosphodiesterase activity. Therefore, the *in vivo* agglutination phenotype results correlated with the *in vitro* phosphodiesterase activity result. In addition, the purified FimY protein, a positive regulator of type 1 fimbriae, also did not demonstrate such activity. Our results indicated that STM0551 has PDE activity *in vitro*. Currently, we can only say that *stm0551* takes part in the complicated type 1 fimbrial regulatory network and play a repressive role. We have no direct evidence about whether *stm0551* actually modulates the concentration of the c-di-GMP pool in *S*. Typhimurium to achieve its impact on *fim* gene regulation. Although the determination of the intracellular concentration of c-di-GMP of Δ*stm0551* mutants warrants further investigation, this may be prove to be difficult because the c-di-GMP concentration fluctuates locally, due to the spatial compartmentalization of proteins [32]. One example of this phenomenon is that the majority of the c-di-GMP in *Acetobacter xylinum* is bound by a membrane protein and is released only in response to certain signals [33]; therefore we need to take into consideration that the actual and measured concentrations of c-di-GMP might be different.

Besides fimbrial production, it is interesting to investigate whether *stm0551* can influence other phenotypes of *S*. Typhimurium. We tested the ability of bacteria to form biofilm, swimming and swarming motility, and the ability to bind Congo red (rdar morphotype) in the LB5010 and Δ*stm0551*strains, but both strains exhibited the same phenotype [34,35] (data not shown). In summary, our study has suggested for the first time that *stm0551* allele which encodes a PDE, play a regulatory role in the production of type 1 fimbriae in *S*. Typhimurium.

## Conclusions

The c-di-GMP pathway is used by most bacteria (but not eukaryotes or Archaea) to regulate numerous biological processes [36]. Several lines of evidence have indicated the concentration of c-di-GMP, balanced by diguanylate cyclase (DGC) and phosphodiesterase (PDE), account for the fimbrial regulatory network in some microorganisms. In *S*. Typhimurium, it has been demonstrated that production of curli fimbriae was inhibited by a PDE STM3611[18]. However, no other type of fimbrial expression in this microorganism has thus far been shown to be controlled by DGC or PDE. The present study revealed that a previously uncharacterized *stm0551* gene, which could encode a PDE, contributes to the down-regulation of type 1 fimbrial expression in *S*. Typhimurium. Our finding may provide valuable information that may help to further elucidate the complicated type 1 fimbrial regulatory circuit in this pathogen.

## Methods

### Bacterial strains, plasmids, and culture media

The bacterial strains, plasmids, and primers used in the present study are listed in Table 1 and Table 2. The *S*. Typhimurium strain used was LB5010, an LT2 derivative [21]. This strain produces type 1 fimbriae and has a variable fimbrial phase. Bacteria were cultured in Luria-Bertani (LB) broth (Difco/Becton Dickinson, Franklin Lakes, NJ) or plated on LB agar. When required, media were supplemented with antibiotics at the following concentrations: 100 μg/ml ampicillin, 50 μg/ml kanamycin, and 20 μg/ml chloramphenicol. Antibiotics were obtained from Sigma (St. Louis, MO). To detect gene expression, 1 mM of isopropyl-β-D-thiogalactopyranoside (IPTG) was used (MDbio, Taipei, Taiwan).

### Construction of a *S*. Typhimurium *stm0551* mutant

A *stm0551* mutant was created by one-step gene inactivation method as described previously [20]. Briefly, a kanamycin-resistance gene from pKD13 tagged with a flanking sequence of the *stm0551* gene was generated by a polymerase chain reaction (PCR) technique. The designed nucleotide sequence was generated with *Pfu* polymerase (Fermentas, St. Leon-Rot, Germany) on a GeneAmp PCR system 2700 thermal cycler (Applied Biosystems, Foster City, CA) and initially incubated at 94 ° C for 3 min, followed by 30 cycles of 94°C for 1 min, 50°C for 1 min, and 72°C for 2 min. Primers used in this approach are listed in Table 3. Then, the PCR product was introduced by electroporation into *S. enterica* serotype Typhimurium LB5010 possessing the pKD46 plasmid which expressed λ Red recombinase [20]. All transformants were grown on LB agar containing kanamycin. The constructed mutants were verified by PCR with primers located in the flanking sequence of the *stm0551* gene.

### Yeast agglutination and guinea pig erythrocyte hemagglutination test for type 1 fimbriae

Tested bacteria were cultured in static LB broth at 37°C for 48 h or on LB agar at 37°C overnight. Bacterial cells in static-broth medium were collected by centrifugation, and the pellet was resuspended in 100 μl of 1× phosphate-buffered saline (PBS). Bacteria from LB agar were scraped with a sterile loop and resuspended in 300 μl of 1× PBS. Subsequently, 30 μl of a 3% (vol/vol) suspension of *Saccharomyces cerevisiae* (Sigma) or guinea pig red blood cells in PBS and an equal amount of bacterial cells to be tested were mixed on a glass slide [27]. Visible agglutination after gentle agitation indicated a positive reaction for type 1 fimbriae. The presence of mannose-sensitive yeast cell agglutination or mannose-sensitive guinea pig erythrocyte hemagglutination was determined by mixing the bacterial suspension with PBS containing 3% (w/v) α-methyl-D-mannoside (Sigma).

### Electron microscopy

The bacterial strains tested were grown in static broth or on solid agar and resuspended in 1 × PBS. The bacterial cells were then negatively stained with 2% phosphotungstic acid and observed with a Hitachi H-600 transmission electron microscope (Hitachi Ltd., Tokyo, Japan).

### Complementation test

Primers used for the complementation test (stm0551-F and stm0551-R) are listed in Table 2 and were used to amplify genomic DNA of *S*. Typhimurium LB5010. The PCR product that possessed the full coding sequence of *stm0551* was cloned into the pACYC184 vector using T4 DNA ligase (Fermentas). To construct a *stm0551* allele with the glutamic acid at position 49 replaced with an alanine; stm0551-F and E49A-TOPO-R were used to amplify the first DNA fragment using *Pfu* DNA polymerase (Fermentas). The PCR conditions were: denaturing at 94°C for 3 min followed by 35 cycles of 94°C for 45 sec, 50°C for 45 sec and 72°C for 45 sec. The second DNA fragment was amplified using E49A-TOPO-F and stm0551-R with the same procedure described above. These two DNA fragments were purified by Montage Gel Extraction Kit (Millipore, Billerica, MA). Ligation of these two DNA fragments having two overlapping ends was achieved with stm0551-F and stm0551-R primers as follows: denaturation at 94°C for 3 min, ligation at 50°C for 45 sec and elongation at 72°C for 45 sec, followed by 35 cycles of 94°C for 45 sec., 50°C for 45 sec, and 72°C for 45 sec. Amplified DNA fragment was digested with *Bam*HI and *Eco*RV and cloned into pACYC184 vector to generate pSTM0551E49A. The mutated *stm0551* allele of this plasmid was sequenced to confirm if the glutamic acid (E) at position 49 was replaced by alanine (A) before transforming into the *S*. Typhimurium Δ*stm0551* strain by electroporation. The pACYC184 cloning vector was also transformed into the *S*. Typhimurium Δ*stm0551* strain as a control.

### Quantitative RT-PCR analysis

Total bacterial RNA was isolated using an RNeasy Mini Kit (Qiagen, Hilden, Germany) according to the manufacturer’s protocol. Subsequently, RNA was treated with RNase-free DNase (1 unit/1 μg RNA) to remove contaminating genomic DNA. The purified RNA was converted to cDNA using a MMLV reverse transcriptase method in the following steps: First, 100 ng of the total RNA was annealed with 100 nM specific primers by heating to 65°C for 2 min and then cooled on ice for 1 min. Next, 1 U of RNasin, 2 μl of 100 mM DTT, 1 μl of 10 mM dNTP and 0.5 μl of 200 U/μl MMLV High Performance Reverse Transcriptase (Epicentre, Madison, WI) were added to each RNA/primer mixture and incubated at 37°C for 1 h, followed by heating at 85°C for 10 min to inactivate the enzyme and then chilled on ice for at least 1 min. The specific cDNA that we prepared was used in the following quantitative real-time PCR analysis. The components of real-time PCR were prepared by adding 10 ng of each specific cDNA and 1 μl of a 10 mM primer solution to 2 × Maxima SYBR Green/ROX qPCR Master Mix (Fermentas) and adjusted with ddH_2_O to a final volume of 20 μl. Cycling conditions were performed using Roche LightCycler 2.0 system (Roche Applied Science, Branford, CT) as follows: 95°C for 2 min followed by 40 cycles of 95°C for 30 sec, 50°C for 30 sec and 72°C for 15 sec. Dissociation curves and non-template controls were included to detect any primer dimerization or other artifacts. The mRNA transcript levels were obtained by the method described by Livak and Schmittgen [37].

### Fusion protein construction

A carboxy terminal 6 × histidine-tagged fusion to STM0551 was constructed by amplifying *stm0551* with primers stm0551-TOPO-F and stm0551-TOPO-R using genomic DNA of *S*. Typhimurium LB5010 as the template. The resulting 316-bp PCR product was cloned into the pET101/D-TOPO vector (Invitrogen, Carlsbad, CA) giving rise to plasmid pSTM0551-His. This recombinant plasmid was sequenced at the adjacent portion of the cloning site to make sure it was in frame before subsequent transformation step. BL21Star™ (DE3) One Shot® chemically competent *E*. *coli* (Invitrogen) cells were transformed with pSTM0551-His. Log phase cultures were induced to express STM0551-His by adding 1 mM IPTG at 37°C for 4 hr. The STM0551-His fusion protein was further purified by ProBond purification kit (Invitrogen) using the protocol provided by the manufacturer. The protein concentration was determined using the Bradford reagent (Fermentas) [38]. A mutant allele of *stm0551* was constructed by site-directed mutagenesis using overlapping-extension PCR of *S*. Typhimurium LB5010 strain genomic DNA template and mutagenic oligonucleotides E49A-TOPO-F and E49A-TOPO-R [39]. Briefly, STM0551-TOPO-F and E49A-TOPO-R were used to amplify the first DNA fragment using Pfu DNA polymerase (Fermentas). The PCR conditions were: denaturing at 94°C for 3 min followed by 35 cycles of 94°C for 45 sec, 50°C for 45 sec and 72°C for 45 sec. The second DNA fragment was amplified using E49A-TOPO-F and STM0551-TOPO-R with the same procedure described above. These two DNA fragments were purified by Montage Gel Extraction Kit (Millipore, Billerica, MA). Ligation of these two DNA fragments having two overlapping ends was achieved with STM0551-TOPO-F and STM0551-TOPO-R primers as follows: denaturation at 94°C for 3 min, ligation at 50°C for 45 sec and elongation at 72°C for 45 sec, followed by 35 cycles of 94°C for 45 sec., 50°C for 45 sec, and 72°C for 45 sec. Amplified fragments were cloned into pET101/D-TOPO vector and sequenced to determine if the glutamic acid (E) at position 49 was replaced by alanine (A). The resulting recombinant plasmid was designed as pSTM0551E49A-His. Further protein induction and purification were performed using the same procedure as for STM0551-His fusion protein. Similarly FimY-His fusion protein was constructed using fimY-TOPO-F and fimY-TOPO-R primers.

### PDE activity assay

*In vitro* PDE activity assays were performed using purified STM0551-His, STM0551E49A-His and FimY-His proteins. Test protein was suspended in the assay buffer (50 mM Tris–HCl and 1 mM MnCl2, pH 8.5) supplemented with 5 mM bis (*p*-nitrophenol) phosphate (bis-*p*NPP) as previously described [40,41]. Reactions were incubated at 37°C overnight. The release of *p*-nitrophenol was quantified at OD_410_ in a spectrophotometer (WPA Biowave II, Cambridge, UK).

### Statistical analysis

All statistical data were analyzed using Student’s *t*-test. Differences in measurements with a *p* value of < 0.05 were considered to be significant.

## Authors’ contributions

The authors have no competing interests. K.-C. Wang drafted the manuscript and performed mutant strain construction and PDE assay. Y.-H. Hsu helped the experimental design and data analysis. Y.-N. Huang assisted molecular cloning and site-directed mutagenesis, protein purification experiments. K.-S. Yeh conceived and coordinated this study and also helped to draft the manuscript. All authors read and approved the final manuscript.
